# Erratum

**DOI:** 10.1093/icvts/ivad125

**Published:** 2023-08-02

Upon original publication, the below-listed manuscripts were included into Volume 36, Issue 7, instead of Volume 37, Issue 1 of *Interdisciplinary CardioVascular and Thoracic Surgery*.

The Publisher apologizes for this error, which has now been corrected.

https://doi.org/10.1093/icvts/ivad070

https://doi.org/10.1093/icvts/ivad112

https://doi.org/10.1093/icvts/ivad113

